# Novel quantitative and specific RT-qPCR assay for UK subtypes of European strain of Tick-borne encephalitis virus

**DOI:** 10.1016/j.virusres.2025.199658

**Published:** 2025-11-04

**Authors:** Mollie Curran-French, Jake D’Addiego, Stuart Dent, Gillian Slack, Kyle Perrins, Fern Jenkins, Nyah Davis, Roger Hewson

**Affiliations:** a UKHSA, Porton Down, UK; b University of Liverpool, HPRU-EZI, UK; c London School of Hygiene and Tropical Medicine, UK

**Keywords:** RT-PCR, Tick-borne encephalitis, Louping Ill virus, Flavivirus, Tick-borne disease

## Abstract

Two closely related tick-borne *Orthoflaviviruses* have been detected in the UK; tick-borne encephalitis virus (TBEV) and louping ill virus (LIV). Diagnosis of tick-borne encephalitis is achieved by detection of IgM/IgG antibodies using ELISA, Immunofluorescence, or by RT-PCR. High sequence and structural similarities between these viruses means they are hard to distinguish due to high cross-reactivity. We have developed a novel RT-qPCR assay using recently identified subtypes of TBEV in the UK and a probe incorporating locked nucleic acids, strengthening hybridisation between the probe and target RNA/DNA, resulting in greater discrimination between thermodynamically similar sequences. Cell-cultured virus extracts and an in vitro transcript of the NS1 gene were used for assay validation. TBEV was specifically detected to a sensitivity of 13 copies per reaction at 95 % confidence with 94 % efficiency. The assay showed no cross-reactivity to 10 Flavivirus RNA extracts tested. Of 43 clinical tick bite samples tested, 23 were TBEV seropositive and all tested negative by the currently used qPCR assay, matching our assay results. A sensitive, highly specific RT-qPCR assay that distinguishes between UK subtypes of TBEV and LIV. This distinction is vital for both accurate clinical diagnosis and vector surveillance, especially in the UK where both viruses co-circulate and cross-reactivity remains a significant diagnostic challenge.

## Highlights

• Tick-borne encephalitis cases are increasing in the UK.
• Louping ill virus is a closely related *Orthoflavivirus* endemic to the UK.
• TBEV UK strains are indistinguishable from LIV by current PCR assays.
• A novel qPCR specific to UK strains of TBEV was developed using locked nucleic acid probes.
• qPCR was able to differentiate between UK subtypes of TBEV and LIV.

## 1 Introduction

Tick-borne encephalitis virus (TBEV) is a member of the *Flaviviridae family* and one of many important human pathogens within the *Orthoflavivirus* genus. Such pathogens include mosquito-borne viruses like yellow fever virus (YFV), dengue virus (DENV), Zika virus (ZIKV) West Nile virus (WNV), Japanese encephalitis virus (JEV) and tick-borne viruses such as louping ill virus (LIV), Omsk haemorrhagic fever virus (OHFV), Kyasanur Forest disease virus (FDV) and Powassan virus (POWV) (International Committee on Taxonomy of Viruses (ICTV) 2025). TBEV is the etiological agent of tick-borne encephalitis (TBE), a disease of the central nervous system spread by ticks of the genus *Ixodes*. TBEV is a small spherical enveloped virus around 50 nm in diameter with a single-stranded positive-sense RNA genome of approximately 11 kb encoding 3 structural proteins (capsid, membrane, and envelope proteins) and 7 non-structural proteins (Fuzik et al., 2018). The genome is translated from a single open reading frame, producing a polyprotein that is cleaved co and post-translationally by host and viral proteases (Ruzek et al., 2019).

TBEV is endemic across Europe and Asia with 3650 cases diagnosed in European and European-associated countries in 2022 (Tick-borne encephalitis Annual Epidemiological Report for 2022 ECDC 2022). The three main subtypes of TBEV; Far-eastern, Siberian and European, have differing pathogenicity, with two additional subtypes having been reported more recently, namely the Baikalian and the Himalayan subtypes (Demina et al., 2010). The majority of TBEV infections of the European subtype are asymptomatic, however of the symptomatic cases an estimated 13–44 % have CNS involvement (Bogovic et al., 2022). European subtype of TBEV infections typically follow a biphasic disease course, with the initial viraemic stage involving non-specific influenza-like symptoms such as fever, muscle aches and malaise followed by the secondary phase involving encephalitis, meningitis and meningoencephalitis (Kaiser, 2008).

There are no anti-viral therapies available to treat TBEV; however, two whole inactivated virus vaccines are licenced in Europe and Canada, which have been shown to protect against severe disease (Kubinski et al., 2020). Countries such as Austria and Latvia have successfully introduced vaccination campaigns reaching vaccine uptake rates of 80 % (Pilz et al., 2023).

TBEV was first detected in the UK following a deer sero-surveillance study which informed the subsequent collection of *Ixodes* ticks in areas with reported seropositivity between 2018–19 (Holding et al., 2019). As of 2023, one confirmed human case and 2 probable human cases have been reported (Kreusch et al., 2019; Tick-borne encephalitis detection in England 2023). Recent tick surveys suggest a possible expansion in *Ixodes* tick populations in the UK which may increase the risk of TBEV infections, as people’s lifestyles are changing to include increased time spent in outdoor settings (Gandy et al., 2023).

Diagnosis during early infection is important to rule out other tick-associated illnesses such as borreliosis and rickettsiosis which exhibit similar flu-like symptoms. Currently, the diagnostic RT-PCR assay used to detect TBEV in the UK is the assay published by Schwaiger and Cassinotti (2003) which amplifies European strains of TBEV. However, this assay also amplifies LIV (Fig. 6A), which is problematic for the UK where both viruses circulate.

LIV is a closely related *Orthoflavivirus* that shares high genome sequence similarity with contemporary European TBEV strains, with approximately 85 % nucleotide identity. LIV is found across the UK, particularly in upland areas with grazing sheep (Jeffries et al., 2014). Infections are mostly associated with sheep and red grouse. The disease in sheep has a mortality rate of 5–60 % depending on age and exposure history, with most fatal cases occurring in lambs with no protective antibodies from their mothers (Jeffries et al., 2014). A formalin-inactivated vaccine which was approved for animal use in the 1970s, has been discontinued in recent years (Reid et al., 1972).

Human infection with LIV, although rare, has been documented in over 44 cases since 1934, primarily among animal handlers, veterinarians and laboratory workers (Jeffries et al., 2014; Davidson et al., 1991). The clinical presentation is variable; infections can be asymptomatic, present as a single febrile phase or - less commonly - follow a biphasic course with neurological involvement including encephalitis (Davidson et al., 1991). While most of the reported cases were in immunocompetent individuals, the possibility of enhanced severity in immunocompromised hosts has not been formally studied. In a notable 2013 case, a 29-year-old woman who presented with refractory seizures following occupational exposure to sheep was presumed to have a LIV infection and had positive flavivirus serology in the absence of other diagnoses (Walkington et al., 2013). Diagnosis in most human cases has relied on serological testing, but due to the high cross-reactivity with TBEV, definitive confirmation of LIV as the causative agent remains difficult in the absence of virus isolation or TBEV exclusion by specific molecular testing. LIV is detected by PCR using the assay developed by Marriott et al. (2006); however, UK TBEV isolates are also amplified, thus this assay cannot be used for definitive diagnosis of UK-acquired LIV infections (Fig. 6B).

Sero-cross-reactivity between flaviviruses is also a notorious diagnostic problem, due to the high genome sequence and structural similarities between various species (Chan et al., 2022), exacerbated by the fact that many areas are endemic with two or more closely related flaviviruses. Current commercial ELISA and IFA assays are not able to distinguish between these species to a high-specificity (Koraka et al., 2002).

Differential diagnosis of TBEV and LIV is important for both public health and veterinary disease management. Although human infection with LIV is considered rare, with only ∼44 reported cases since 1934 and no confirmed fatalities, the true burden remains uncertain due to limitations in past diagnostic testing and serological cross-reactivity with TBEV. In contrast, European TBEV strains are associated with more frequent neurological disease and have an estimated human case-fatality rate of up to 2 % (Bartholdsson et al., 2025). Accurate detection of TBEV is critical for public health surveillance, informing vaccine strategy, and distinguishing cases from LIV where appropriate.

LIV remains a notifiable livestock pathogen in the UK and causes economically significant outbreaks, particularly in sheep. A veterinary surveillance report from SRUC recently confirmed new cases of louping ill in the Scottish Borders, underscoring the virus's ongoing relevance in animal health (Louping Ill 2025).The discontinuation of commercial animal vaccines against LIV further elevates concern, particularly as rising infection rate in sheep and dogs increase the risk to farmers, veterinarians, and dog owners. Accurate diagnosis and ongoing surveillance of LIV cases in animals are essential for public health risk assessment, zoonotic preparedness and supporting future vaccine development (Tandavanitj et al., 2024).

Thus to discriminate between these viruses, we developed a locked nucleic acid RT-qPCR assay designed to selectively detect European TBEV strains without cross-reacting with LIV. The assay was validated using in vitro transcript standards, cell culture-derived isolates and clinical specimens.

## 2 Materials & methods

### 2.1 Virus strains

Ticks were collected in Hampshire and pooled as previously described (Holding et al., 2019), then homogenised in phosphate buffered saline (PBS). All homogenates were tested on the Schwaiger PCR and any positives were subsequently sequenced and confirmed to be TBEV. One TBEV positive homogenate was inoculated onto A549 cells (ECACC 86012804). After 5 days, cytopathic effect (CPE) was observed and the supernatant was clarified and harvested. The virus was plaque-purified and cultured on A549 cells. The isolated virus was designated TBEV Hampshire subtype (MN661145). Reference samples of TBEV Neudorfl (NC001672.1) and LIV (KP144331.1) were obtained from the National Collection of Pathogenic Viruses (NCPV) and passaged on A549 cells. The supernatant was clarified after 4 days when CPE was observed. Several Flaviviruses were cultured *in vitro* and sequenced to confirm strain and mutations: LIV (KP144331.1), USUV (NCPV 1105081v), WNV (C407666.1), ZIKV (NCPV 1308258v), DENV serotypes 1–4 (NCPV 0106037v, NCPV 0006041v, NCPV 9911281v, NCPV 0411285v), JEV (NCPV 0106036v), RVFV (NCPV 1202191v), ALKV (OP037813.1), KFDV(JF416960.1) and YFV (NCPV 0006042v).

### 2.2 Alignment and primer design

**Fig. 1:**
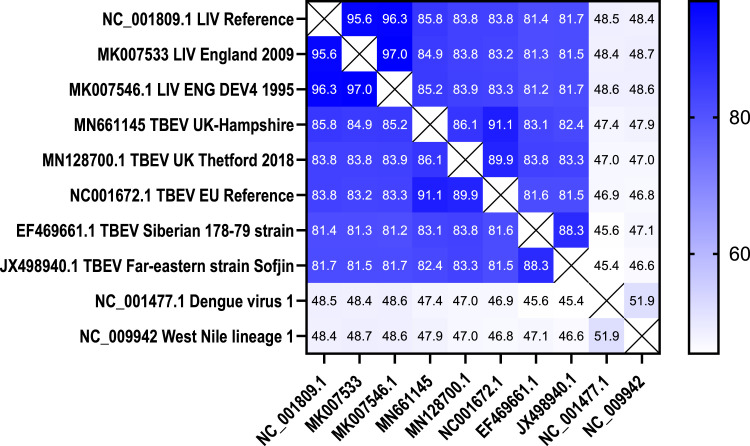
Heat map comparison of nucleotide identities of multiple TBEV and LIV isolates displayed as percentage identity. Darker shades of blue indicate a higher percentage identity, showing a percentage identity of 81–86 % between LIV and TBEV isolates. (For interpretation of the references to colour in this figure legend, the reader is referred to the web version of this article.).

The published sequences of three TBEV isolates, NC001672.1, MN128700.1, MN661145, and three LIV isolate sequences, NC001809.1, MK007533, MK007546.1 were aligned using Geneious prime software with the following settings: free end gaps with a 65 % similarity (5.0/−4.0), a gap open penalty of 12, gap extension penalty of 3 and refinement iterations of 2. (Fig. 1).

To develop an assay that specifically amplifies TBEV while excluding LIV, we performed a comparative alignment of complete genomes representing all known European TBEV and LIV isolates. A short region within the non-structural protein 1 (NS1) gene was identified as having the highest concentration of nucleotide mismatches between the two viruses, this provided an opportunity for sequence-based discrimination. Thus this region was selected for several reasons: NS1 is generally conserved among *Orthoflavivirus* members, which facilitates reliable primer binding and consistent assay performance across strains, however it also contains species- and sup-type specific motifs that enable detailed molecular differentiation (Fisher et al., 2023). Also functionally, NS1 is an essential glycoprotein secreted during viral replication and its been successfully targeted in other validated flavivirus assays such as DENV 1–4 and WNV (Kim et al., 2015; Kauffman et al., 2003), demonstrating its diagnostic utility and stability as a genomic target.

**Fig. 2:**
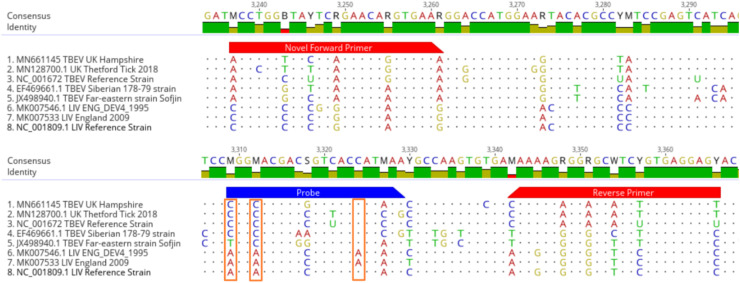
Multiple isolates of TBEV and LIV NS1 sequences aligned using Geneious Prime software set to high sensitivity. The TBEV assay primers and probe binding locations are indicated by the red and blue regions respectively. Nucleotides that are mismatched to the are shown in colour specific to each base and positions of the locked nucleic acids are indicated by the orange boxes in the probe sequence. (For interpretation of the references to colour in this figure legend, the reader is referred to the web version of this article.).

By focussing on a short, diagnostically informative stretch within a conserved gene, we maximised assay robustness while ensuring sufficient genetic divergence from LIV. This balance supports both sensitivity across European TBEV strains and specificity against closely related Orthoflaviviruses. Primers and probes were designed to target the region shown in Fig. 2. Primer-probe interactions were evaluated using the Oligo Analyser tool (Integrated DNA Technologies) and the TBEV probe was labelled with a 5′ 6-carboxyfluorescin (6-FAM) reporter dye, and a 3′ Iowa Black FQ quencher. The probe design incorporated LNA modifications at specific nucleotide positions within the probe sequence, as highlighted in the orange boxes in Fig. 2 and the nucleotides in bold typeface in Table 1. These positions were selected because they are consistently conserved across European TBEV strains but also exhibit mismatches in the corresponding LIV sequences. Incorporating LNA bases at these discriminative positions enhances mismatch discrimination and improves assay specificity by preventing amplification of LIV RNA while maintaining high sensitivity for TBEV. Locked nucleotides include a methylene bridge between the 2′-O and the 4′-C of the ribose ring, with their incorporation into the DNA oligonucleotide probe increasing the pairing stability of a complementary nucleotide strand leading to increased hybridisation temperature (Koshkin and Wengel, 1998) and allowing discrimination between TBEV sequences over LIV sequences. The locked bases also increase the stability of the probes and their resistance to nucleases and allow a shorter probe design which increases signal to noise ratio. The probe was designed avoiding the use of locked nucleic acid on G-T mismatches as it has shown to have less discrimination capability (You et al., 2006).

**Table 1:** Novel Primers and locked base probe used to detect TBEV RNA in clinical samples and cultured virus extract.

	*Sequence 5′−3′*	*Genome position*
*F-TBEV*	*ACCTGGTTACTCAGAACAGGTGAAA*	*3235–3259*^a^
*R-TBEV*	*CAAAAGAGGAGCATCTGTGAGGAGT*	*3340–3364*^a^
*P-TBEV*	56-FAM**/+C**GG**+C**ACGACGGTCAC**+C**ATAAA/3IABkFQ/	*3307–3327*^a^*^b^*

a MN661145 TBEV UK Hampshire sequence positions Locked bases are indicated with *a* + and in bold typeface.

### 2.3 Primers and probes

A louping ill virus PCR assay was used to test the cross-reactivity of UK strains of TBEV and a pan-flavivirus PCR was used to ensure cross-reactivity RNA was not degraded see details in Table 2.

**Table 2:** Primers and Probes used for confirmatory testing from previously published diagnostic TBEV, LIV and Pan-Flavivirus assays.

	*Sequence 5′−3′*	*Genome position*	*References*
*F-TBEV*	*GGG CGG TTC TTG TTC TCC*	*11054-11071*	*NC001672* (*Schwaiger and Cassinotti, 2003*)
*R-TBEV*	*ACA CAT CAC CTC CTT GTC AGA CT*	*11099-11121*	
*P-TBEV*	TGA GCC ACC ATC ACC CAG ACA CA	*11073-11095*	
*F-LIV*	5’ GCT GTC AAG ATG GAT GTG TAC A 3’	*1714–1735*	*NC001809.1* (*Marriott* et al.*, 2006*)
*R-LIV*	5′ ACT TGT TTC CCT CAA TGT GT 3′	*1791–1810*	
*P-LIV*	5′−6FAM CTG GAG TGC TGA A MGB −3′	*1751–1765*^a^	
*F-Flavi*	TACAACATGATGGGGAARAGAGARAA	*Various*	(*Patel* et al.*, 2013*)
*F-Flavi S2*	TACAACATGATGGGMAAACGYGARAA	*Various*	
*R-Flavi AS4*	GTGTCCCAGCCNGCKGTRTCRTC		
*P-Flavi 1*	6FAM-TG**+G**TWYATGT**+G**GYTNG**+G**RGC-BHQ1		
*P-Flavi 2*	6FAM-CCGTGCCATATGGTATATGTGGCTGGGAGC-BHQ1		
*P-Flavi 3*	6FAM-TTTCTGGAATTTGAAGCCCTGGGTTT-BHQ1		

a Mismatch in Probe at 3′ terminal A in probe is a G in the reference sequence.

### 2.4 In vitro transcript control

A DNA fragment of TBEV Hampshire (MN661145) NS1 flanking the PCR target by 319 nucleotides upstream and 324 nucleotides downstream of the forward and reverse primers binding sites respectively was synthesised by Eurofins genomics. The DNA fragment contained a 5′ T7 promoter region and a 3′ SP6 promoter sequence. The DNA transcript sequence was amplified using T7 and SP6 primers and the Q5 high-fidelity polymerase (M0491S). The size of the PCR product was confirmed by electrophoresis using a 1 % agarose gel and purified using the QIAquick PCR purification kit (Qiagen 28104). RNA was transcribed using the HiScribe T7 High yield RNA synthesis kit (NEB E2040S). Briefly, DNA was incubated with T7 polymerase at 37C for 2 h followed by DNAase I (NEB 0303) treatment to remove residual DNA. RNA was then purified using RNeasy kit (QIAGEN 74104) as per manufacturer’s instructions and quantified using Qubit broad range and high sensitivity assay kits (Invitrogen- Q32852, Q10210). The IVT RNA was serially diluted 10-fold from 5000,000 copies to 5 copies per reaction and ran with PCR primers to check the efficiency of the reaction which was 97 % (Fig. 4B).

### 2.5 Viral RNA extraction

All viral RNA was extracted from virus culture stocks and clinical specimens by adding 140ul sample to 560ul AVL and a subsequent 560ul Ethanol. RNA was then extracted using QIAamp viral RNA Mini kit (Qiagen 52904) according to the manufacturers protocol.

### 2.6 RT-PCR assay

The RT-qPCR was performed using TaqMan Fast Virus 1-Step master mix (Applied Biosystems 4444432) which contains Moloney Murine Leukemia Virus Reverse Transcriptase and AmpliTaq Fast DNA Polymerase for use in a single reaction. TBEV RNA was amplified in total 20ul reactions with 1X Fast virus master mix, 100 nm TBEV primers, 50 nm TBEV probe, 7.5ul of RNase free water and 5ul of RNA. The cycling conditions included a reverse transcription step for 5 mins at 50 °C and an initial denaturation step at 95 °C for 20 s, followed by 40 cycles of a denaturation step at 95 °C for 3 s and an anneal/extension step at 55 °C for 30 s. The extension/annealing temperature was chosen based on the predicted primer/probe melting temperatures. Quantification of RNA was achieved using our in vitro transcript from 5 to 5 000 000 copies per reaction. All runs were performed on the Applied Biosystems Viia7 instrument. The lower limit of detection was calculated using GraphPad prism software by diluting the TBEV IVT from 40 copies to 0.3 copies per reaction with 9 replicates per concentration by 2 different users.

## 3 Results

### 3.1 Optimisation of primer/probe concentrations

**Fig. 3:**
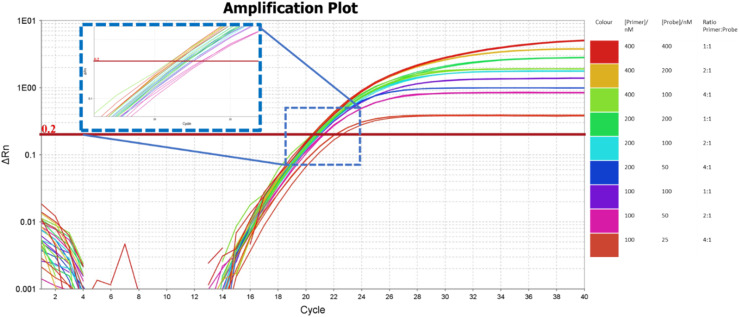
PCR Amplification plot using different primer/probe concentrations and ratios to identify the most efficient amplification reaction. Primers and Probes were tested at the following concentrations 400:400 nM (red), 400:200 nM (orange), 400:100 (light green), 200: 200 nM (green), 200:100 nM (light blue) 200:50Nm (blue), 100:100 nM (purple), 100:50 Nm (pink), 100:25 Nm (pastel red). The blue box represents a zoomed-in image of the point at which the amplification reactions cross the threshold. (For interpretation of the references to colour in this figure legend, the reader is referred to the web version of this article.).

Primers at various concentrations (400/200/100/50 nM) and probes (400/200/100/50/25 nM) at different ratios (1:1, 2:1, 4:1) were tested at 50 000 copies per reaction. The reactions with a primer to probe ratio of 4:1 had the lowest delta Rn, crossed the threshold last and therefore were not considered further. Reactions that plateaued between 1–10 delta Rn were considered further. All amplification reactions except those with 100 nM primer concentrations crossed the 0.2 threshold between cycle 20–21 (Fig. 3). Due to cost considerations the primer/probe concentration of 200 nM /100 nM were used for all further experiments.

### 3.2 Sensitivity of assay

Sensitivity of the assay was determined by diluting TBEV IVT from 5 000 000 copies per reaction to 500 copies per reaction with 5 replicates repeated over 3 plates. The correlation coefficients fell between 0.990 and 1. Negative controls containing all reaction mix and water were included on each run with no false positive results recorded.

**Fig. 4:**
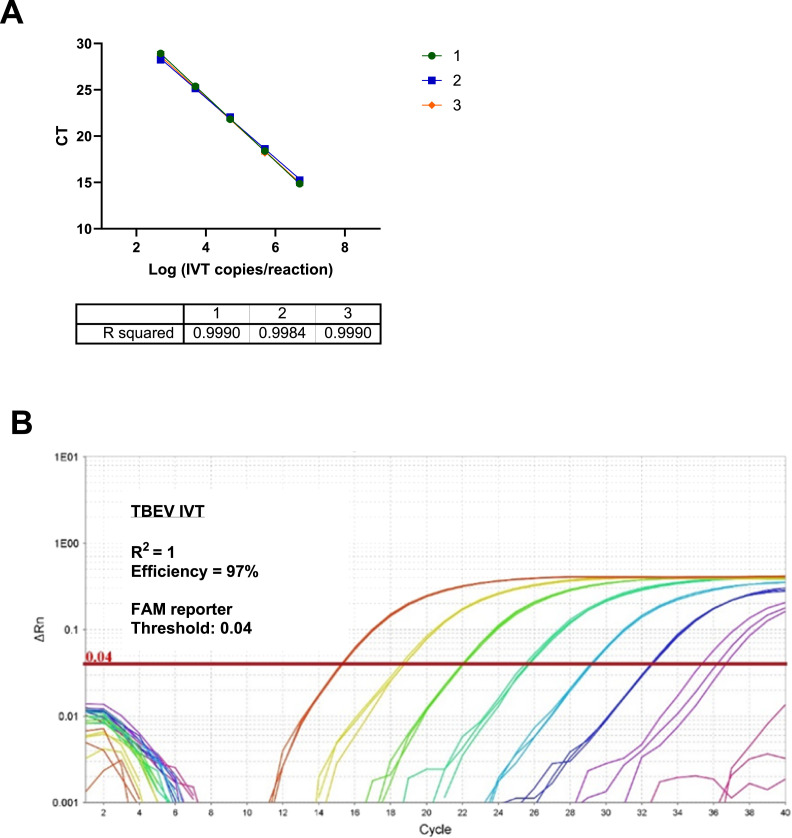
Analytical sensitivity and reproducibility of the TBEV-specific RT-qPCR assay. In vitro transcribed (IVT) TBEV RNA was serially diluted from 5000,000 to 500 copies per reaction. Each dilution was tested in five replicate reactions, and the experiment was repeated three times to assess reproducibility and linearity. A) CT values were plotted against Log IVT copy number per reaction (x-axis) for each of the three replicate experiments. All runs showed consistent linear amplification over a dynamic range of 4–5 log B) Amplification plot of relative fluorescence (delta Rn) vs cycle number for the IVT standard curve using TBEV IVT serially diluted at 5 × 10^7^copies/rxn (red), 5 × 10^6^c/rxn (Yellow), 5 × 10^5^copies/rxn (light green), 5 × 10^4^ copies/rxn (dark green), 5 × 10^3^copies/rxn (light blue), 5 × 10^2^copies/rxn (dark blue), 5 × 10^1^copies/rxn (purple), Negative (pink). The reaction demonstrate consistent exponential amplification and the assay exhibited high linearity (R²=1) and amplification efficiency (∼97 %) across all runs, supporting nice quantitative performance. (For interpretation of the references to colour in this figure legend, the reader is referred to the web version of this article.).

To determine the efficiency of the reaction, TBEV IVT was serially diluted 10-fold from 5 000 000 to 500 copies per reaction. The correlation coefficients shown in Fig. 4A were between 0.998–0.999 and the efficiency between 93 %−101 %, with a representative amplification curve shown in Fig. 4B.

**Fig. 5:**
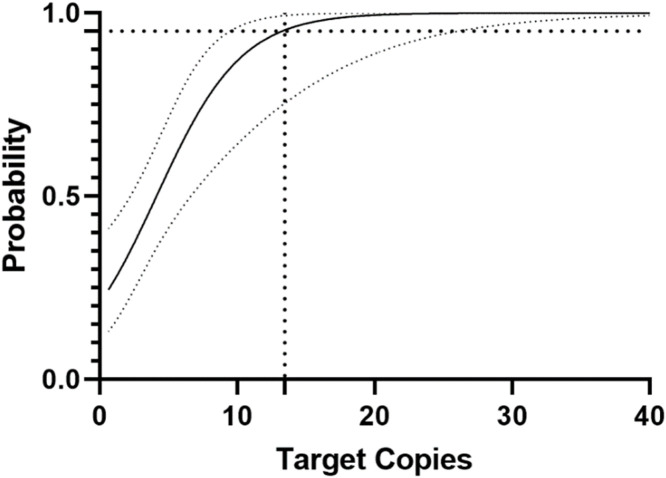
Probit analysis to determine the lower limit of detection at 95 % confidence. Target copies is plotted against the probability of detection. The larger dotted lines show a 95 % probability of detecting TBEV RNA in a sample with 13 copies per reaction. Probit analysis was performed using the GraphPad Prism 10 software using the simple logistic regression analysis.

The lower limit of detection (LLOD) was determined using the IVT diluted from 40 to 0.3 copies per reaction. Each dilution was tested with 9 replicates by two users. Probit analysis revealed a lower limit of detection of 13 copies/reaction at the 95 % confidence level (Fig. 5).

### 3.3 Reproducibility of assay

#### 3.3.1 Intra and inter-assay variability

Intra-assay variation was assessed by running 12 replicates of 3 concentrations of 500 000, 50 000, 5 000 TBEV IVT copies across a plate. The coefficient of variance (CV) was calculated as 0.67, 0.41 and 0.37, respectively. Inter-assay variation was assessed by running the TBEV IVT at the same three concentrations as 5 replicates on 5 different days. The percentage CV was 1.02, 0.44, 5.32 for 500 000, 50 000 and 5 000 (Table 3). The higher variation seen at 5 000 copies per reaction can be attributed to greater influence of stochastic effects at lower copy numbers.

**Table 3:** Coefficients of variance expressed as percentage calculated using CT values.

	Copies of TBEV IVT		
	500 000	50 000	5 000
Intra-assay variation (CV %)	0.67	0.41	0.37
Inter-assay variation (CV %)	1.02	0.44	5.32

#### 3.3.2 Dynamic range

**Fig. 6:**
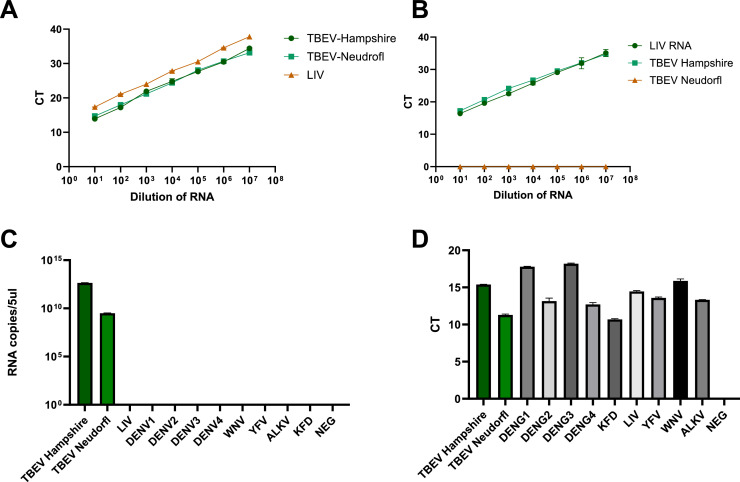
Evaluation of cross-reactivity of Novel European TBEV PCR assay. A) Amplification of TBEV and LIV RNA using the Schwaiger (Schweiger et al. 2003) TBEV RT-PCR assay. Dilution of RNA is plotted against CT. TBEV Hampshire, Neudorfl and Louping ill virus RNA was detected at all dilutions tested. B) Amplification of TBEV Hampshire RNA using a published LIV-specific PCR assay (Marriott et al., 2006). Dilution of RNA is plotted against Ct. The LIV targeting RT-PCR assay designed by Marriott et al. was tested in triplicate against LIV, TBEV Hampshire and TBEV Neudorfl isolates. C) RNA from cell-cultured viruses; TBEV Hampshire, TBEV Neudorfl, dengue 1–4, Kyasunur forest disease virus, louping ill virus, yellow fever virus, West Nile virus, Alkhurma virus (A) Orthoflavivirus RNA tested using the novel TBEV-specific assay. D)Orthoflavivirus RNA tested on pan-flavivirus PCR assay (31). (For interpretation of the references to colour in this figure legend, the reader is referred to the web version of this article.).

RNA extracted from virus isolations in cell culture from two strains of TBEV, UK Hampshire and Neudorfl, were tested using the novel assay. Both TBEV strains were detected in the novel PCR assay. (Fig. 6A).

### 3.4 Evaluation of cross-reactivity between flaviviruses

The cross reactivity of the assay was tested against several common flaviviruses from cell culture derived extracts including LIV, USUV, WNV, ZIKV, DENV serotypes 1–4, JEV, ALKV, KFDV and YFV. Only TBEV- Neudorfl and UK Hampshire RNA amplified in the reaction all other orthoflavivirus RNA tested did not amplify (Fig. 6C), demonstrating high specificity and no cross-reactivity with other orthoflaviviruses tested. As a control for RNA integrity and viral load, a previously published pan-flavivirus PCR assay was used to verify the integrity of all the RNAs tested (31) results shown in Fig. 6D All orthoflavivirus RNA was detected in the pan-flavivirus PCR assay.

### 3.5 Evaluation of non-specific interactions in clinical samples

Tick bite samples of up to 21 days post tick bite were tested to evaluate non-specific interactions. TBEV seropositive samples were all PCR negative, most likely due to the short viraemic period often occurring before the convalescent phase. This temporal dissociation between the viraemic and antibody-positive phases has been well documented (Holzmann, 2003; Lindquist and Vapalahti, 2008; Dumpis et al., 1999). All 43 clinical samples tested negative using the novel TBEV qPCR assay, indicating no non-specific amplification of other clinically relevant infections from tick bite. Of 43 clinical serum and cerebrospinal fluid (CSF) samples tested all were TBEV PCR negative and 23 were TBEV seropositive by IFA and 5 Lyme positive by ELISA (Table 4).

**Table 4:** Screening of acute tick bite samples in the UK by novel European TBEV qPCR.

Antibody detection	TBEV qPCR Pos	TBEV qPCR Neg
TBEV and Lyme-Negative	0	15
Lyme Positive	0	5
TBEV Positive	0	23
Total		43

## 4 Discussion

Although the incidence of TBEV is low in the UK, recent detections of TBEV RNA in ticks and a small number of autochthonous human cases confirm that the virus is now established in multiple geographic foci (Callaby et al., 2025). This expansion aligns with broader trends observed across Europe, where factors such as rising global temperatures, expansion of tick populations, urbanisation and lifestyle changes favouring outdoor activities have facilitated TBEV to spread into previously unaffected regions (Holding et al., 2019; Randolph, 2010; Louping Ill 2022). Although disease burden in the UK is low, identification of multiple enzootic foci and evidence of human exposure underscore the need for robust surveillance systems and accurate diagnostic tools. This study highlights the limitations of current serological and molecular assays in discriminating TBEV from closely related LIV, and presents a novel RT-PCR assay with enhanced specificity for European TBEV strains.

The standard diagnostic approaches in the UK rely on serological detection of IgM and IgG by ELISA in serum or CSF, which is hampered by the delayed IgM seroconversion and substantial cross-reactivity with LIV and other orthoflaviviruses (Louping Ill 2022). This cross-reactivity is particularly problematic in regions where TBEV and LIV co-circulate, making it difficult to distinguish between infections without other confirmatory testing. This issue is further compounded by the discontinuation of the LIV vaccine for animals, which may lead to increased LIV circulation and greater likelihood of exposure in enzootic regions. As a result, there is an even more urgent need for a specific molecular assay that can accurately differentiate TBEV from LIV, both in clinical diagnostics and surveillance efforts. Additionally, serological assays alone cannot provide a definitive diagnosis during the early phase of infection when viremia may be present but an antibody response has not yet developed. These limitations highlight the need for molecular assays capable of early and specific detection.

The RT-qPCR assay developed in this study directly addresses these diagnostic gaps by incorporating locked nucleic acid (LNA) probes. The assay is highly specific to European TBEV while avoiding amplification of LIV and other non-targeted orthoflaviviruses. The importance of this specificity is highlighted in recent findings that the commonly used LIV (Marriott) assay cross-reacts with TBEV Hampshire (Fig. 6B), thereby undermining its utility in differentiating between the two viruses. The assay’s analytical sensitivity falls within the range of viral RNA loads typically observed during early phase of infection (Patel et al., 2013), allowing for timely diagnosis that can inform clinical and public health interventions.

In addition to its clinical utility, the assay is well-suited for use in vector surveillance. Molecular detection of TBEV in ticks is critical for identifying new foci of virus circulation and assessing the zoonotic risk posed to human populations. Importantly, our assay enables rapid and specific detection of European strains of TBEV in tick samples before confirmatory sequencing, which is often resource and time-intensive. As such, it offers a practical tool for integration into routine tick surveillance workflows and can support risk-based decision-making regarding vaccine policy and public health messaging.

One limitation of this study is the inability to empirically evaluate the assay against Far-eastern and Siberian subtypes of TBEV due to the lack of access to these strains and the requirement for biosafety level 4 containment. Nevertheless, broader surveillance applications in regions with mixed subtype circulation would require additional primer and probe sets tailored to other TBEV lineages. Additionally, we did not have access to PCR‑positive clinical material during this study, which restricted our ability to validate the assay on confirmed TBEV-positive human or animal samples. In the UK context, the rarity of TBEV infections and the short duration of viraemia limit the availability of PCR-positive clinical samples for large-scale validation. Nevertheless, the assay’s specificity was confirmed using a panel of 43 clinically relevant, post-tick-bite samples that were representative of routine UK diagnostic submissions. Several precedent studies developing molecular assays for arboviruses proceed with relatively small clinical cohorts or none at all, relying on synthetic standards, cultured virus, and representative panels. For example, Schwaiger & Cassinotti (Schwaiger and Cassinotti, 2003) developed a TBEV qPCR primarily using viral standards; Ayers et al. (Ayers et al., 2006) described a RT-PCR for mosquito-borne flaviviruses using a small field sample set. These precedents support our approach under constrained clinical sample availability. However, comprehensive in silico analysis (Fig. 1; Fig. 2) using publicly available sequences demonstrated multiple mismatches between the assay's enhanced probe region and these non-European strains and we are actively collaborating with surveillance laboratories to secure PCR-confirmed clinical material for future validation.

Moreover, the assay's potential for scalability and validation across multiple diagnostic laboratories could make it an important cornerstone of national and regional diagnostic protocols. The introduction of such a robust diagnostic tool can also drive public health messaging, ensuring that both the public and clinicians are well-informed about the true prevalence and risk of TBEV in the UK.

The design and effectiveness of this assay provides a template for developing diagnostics for other emerging flaviviruses, such as Usutu virus and West Nile virus (Folly et al., 2020), which may pose similar challenges due to cross-reactivity in regions where arthropod vectors are expanding. As such this assay is not just a solution for current challenges but a forward-looking tool in anticipation of future public health needs.

In conclusion, this study provides a timely and practical solution to a critical diagnostic need in the UK. The European TBEV-specific RT-qPCR assay used in conjunction with current serological assays enhances diagnostic accuracy, supports early clinical diagnosis, and strengthens surveillance capability. Its implementation in public health settings will improve case identification, reduce misclassification of LIV infections, and contribute to more accurate risk assessments and targeted interventions. As TBEV continues to expand its range in Europe, diagnostic preparedness will be essential to mitigate the impact of this emerging pathogen.

## Declaration of generative AI and AI-assisted technologies in the writing process

During the preparation of this work the authors used Grammerly in order to improve the readability of the article. After using this tool/service, the authors reviewed and edited the content as needed and take full responsibility for the content of the published article.

## CRediT authorship contribution statement

**Mollie Curran-French:** Writing – original draft, Visualization, Validation, Project administration, Investigation, Formal analysis, Data curation, Conceptualization. **Jake D’Addiego:** Writing – review & editing, Formal analysis, Data curation. **Stuart Dent:** Writing – review & editing, Supervision, Conceptualization. **Gillian Slack:** Writing – review & editing, Supervision, Formal analysis, Conceptualization. **Kyle Perrins:** Writing – review & editing, Data curation. **Fern Jenkins:** Writing – review & editing, Data curation. **Nyah Davis:** Writing – review & editing, Data curation. **Roger Hewson:** Writing – review & editing, Supervision, Funding acquisition, Formal analysis, Conceptualization.

## Declaration of competing interest

The authors declare that they have no known competing financial interests or personal relationships that could have appeared to influence the work reported in this paper.

The author is an Editorial Board Member/Editor-in-Chief/Associate Editor/Guest Editor for this journal and was not involved in the editorial review or the decision to publish this article.

## Data Availability

Data will be made available on request.
